# Helical Antifreeze Proteins Have Independently Evolved in Fishes on Four Occasions

**DOI:** 10.1371/journal.pone.0081285

**Published:** 2013-12-06

**Authors:** Laurie A. Graham, Rod S. Hobbs, Garth L. Fletcher, Peter L. Davies

**Affiliations:** 1 Department of Biomedical and Molecular Sciences, Queen’s University, Kingston, Ontario, Canada; 2 Department of Ocean Sciences, Memorial University of Newfoundland, St. John’s, Newfoundland, Canada; National Institute for Medical Research, Medical Research Council, London, United Kingdom

## Abstract

Alanine-rich α-helical (type I) antifreeze proteins (AFPs) are produced by a variety of fish species from three different orders to protect against freezing in icy seawater. Interspersed amongst and within these orders are fishes making AFPs that are completely different in both sequence and structure. The origin of this variety of types I, II, III and antifreeze glycoproteins (AFGPs) has been attributed to adaptation following sea-level glaciations that occurred after the divergence of most of the extant families of fish. The presence of similar types of AFPs in distantly related fishes has been ascribed to lateral gene transfer in the case of the structurally complex globular type II lectin-like AFPs and to convergent evolution for the AFGPs, which consist of a well-conserved tripeptide repeat. In this paper, we examine the genesis of the type I AFPs, which are intermediate in complexity. These predominantly α-helical peptides share many features, such as putative capping structures, Ala-richness and amphipathic character. We have added to the type I repertoire by cloning additional sequences from sculpin and have found that the similarities between the type I AFPs of the four distinct groups of fishes are not borne out at the nucleotide level. Both the non-coding sequences and the codon usage patterns are strikingly different. We propose that these AFPs arose via convergence from different progenitor helices with a weak affinity for ice and that their similarity is dictated by the propensity of specific amino acids to form helices and to align water on one side of the helix into an ice-like pattern.

## Introduction

Organisms exposed to subzero temperatures are at risk of freezing damage. Marine teleost fishes are particularly vulnerable because they typically freeze at a temperature ∼1°C above that of icy seawater. Those fishes that exploit the ice-rich niches near the poles have adapted by synthesizing either small-molecule colligative antifreeze agents such as glycerol and/or non-colligative macromolecular antifreeze proteins (AFPs) [1]–[3]. Following the discovery of antifreeze glycoproteins (AFGPs) in Antarctic notothenioid fishes [4], three additional AFP types, denoted type I [5], [6], type II [7] and type III [8], [9] were discovered in other fishes. All are thought to function by inhibiting ice growth via their irreversible adsorption to the surface of nascent ice crystals [10].

**Figure 1:**
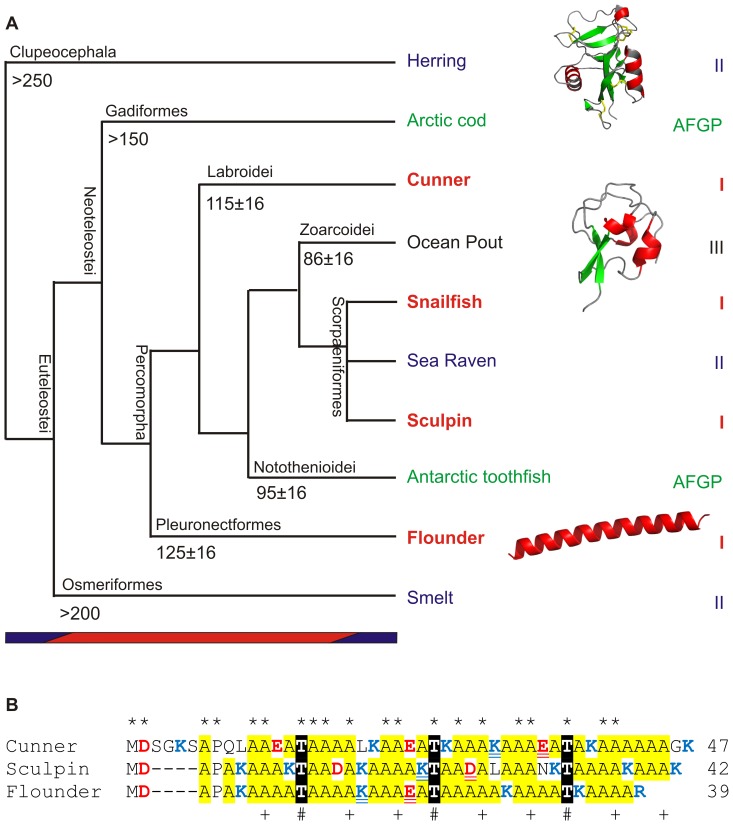
Evolutionary relationships between AFP-producing fishes and the similarities between type I AFPs. A) Phylogenetic relationships (not to scale) among AFP-producing fish from analysis of complete mitochondrial genomes [67]–[69] or selected nuclear and mitochondrial sequences [45]. Estimated divergence times (Ma, some with 95% highest posterior density limits) [45]–[47] are shown at some nodes. Species names are colored by AF(G)P type as indicated on the right. Representative ribbon structures are shown for types II, III, and I AFPs (PDB 2PY2, 1HG7, 1WFA from top to bottom, red = helix, green = strand, gray = coil). The colored bars at the bottom indicate climate differences marked by the presence (blue) or absence (red) of large ice sheets. Common names of representative AFP-producing fish are indicated but their scientific names are as follows; herring (*Clupea harengus*), Arctic cod (*Boreogadus saida*), cunner (*Tautogolabrus adspersus*), ocean pout (*Zoarces americanus*), Atlantic snailfish (*Liparis atlanticus*), dusky snailfish (*Liparis gibbus*), sea raven (*Hemitripterus americanus*), longhorn sculpin (*Myoxocephalus octodecemspinosus*), shorthorn sculpin (*Myoxocephalus scorpius*), Antarctic toothfish (*Dissostichus mawsoni*), winter flounder (*Pseudopleuronectes americanus*) and rainbow smelt (*Osmerus mordax*). B) Alignment of representative type I skin AFPs from three fishes from three separate orders (winter flounder (M63478.1), longhorn sculpin (AF306348.1) and cunner (JF937681.2). Potential or known ice-binding residues within the 11-aa repeat that show an *i*, *i*+4, *i*+8 spacing pattern are indicated with plus symbols (Ala) and number symbols (Thr) with asterisks denoting residues that are identical in all sequences. Acidic and basic residues are in red and blue font respectively, with Ala highlighted yellow and Thr in white font with black highlighting. Potential helix-stabilizing salt bridges consisting of basic and acidic residues with the more effective *i*, *i*+4 separation [70] are double underlined. The cunner isoform is also found in blood [42].

As more fish species were studied, it became apparent that the distribution of several AFP types defied taxonomic conventions (Fig. 1A). The AFGPs and type II AFPs are found in two and three different fish orders, respectively, while helical type I AFPs are found in four superfamilies spanning three orders. Scattered gene distributions such as these can arise by four mechanisms; 1) descent from a common ancestor followed by rampant gene loss in most lineages [11], 2) introgression in which backcrossing of a hybrid introduces new genes into a species [12], 3) convergent evolution in which non-homologous progenitors come to share similarities due to selection (or parallelism if similar changes occur in the same precursor) [13], [14] and 4) lateral transfer in which genes are transferred between different species [15].

The AF(G)Ps of fishes provides a microcosm in which to study the origin and evolution of new genes. Indeed, the presence of four different AF(G)P protein structures in fishes is a clear example of convergent evolution to a common function (functional convergence [11]) of inhibiting the growth of ice. Following its genesis, the distribution of the globular type III AFP, derived from the C-terminal domain of the enzyme sialic acid synthase [16], [17], can be attributed to descent from a common ancestor as it is only found in Zoarcids such as the ocean pout (Fig. 1A). The highly-repetitive AFGP provides a clear example of two additional processes. Loss of AFGP genes has occurred in species of notothenioids that migrated from Antarctica to warmer waters approximately 11 Ma ago [18]. More interestingly, similar AFGPs arose by convergent evolution in two fishes that lie on well-separated phylogenetic branches (Fig. 1A) and reside at opposite poles. Although the progenitor of the cod gene is not yet known, the AFGP of the Antarctic notothenioids is derived from a repetition of a small segment of a trypsinogen gene [19]. Despite the similarities between the proteins, the genes are sufficiently dissimilar to conclude that they arose from different progenitors by convergence [20], which is not unreasonable given that they are composed primarily of variable numbers of simple Ala-Ala-Thr repeats. In contrast, the type II AFPs are more complex globular proteins, derived from lectins, in which both the coding and non-coding sequences from fishes that diverged over 200 Ma ago (herring and smelt) are highly similar. Although gene loss has been proposed as an explanation [21], the greater than 90% identity between several of the introns argues that lateral gene transfer is the only process that can logically explain this similarity [22], [23]. Sea raven also possess a type II antifreeze [24], while the closely-related sculpins produce type I AFPs [25], [26].

It has been suggested that climate change many millions of years ago was the selective pressure that led to the recent acquisition and amplification of the genes encoding AFPs [27]. Most orders and suborders of teleost fish, including those that produce AFPs, diverged during a warm period in earth’s history, from around 250 to 55 Ma (Fig. 1A), when large ice sheets were absent from earth [28]. Continental-scale glaciation was first evident in the Antarctic c. 34 Ma, but the timing and extent of ice formation in the Arctic is still a matter of debate [29]. Nevertheless, sea ice has been a significant factor in the northern oceans over the last three Ma [28] and is surely the selective pressure that fueled the wide range of evolutionary mechanisms observed in the different AFP gene families of northern fishes.

The type I AFPs are widely distributed, being found in four different superfamilies in three different orders. The first to be discovered were from winter flounder (order Pleuronectiformes) in 1974 [5], [6]. More examples followed, from shorthorn sculpin and snailfish (order Scorpaeniformes) [30], [31] and most recently from the cunner (order Perciformes) [32]. Multiple isoforms are now known from a few species. In winter flounder, cDNA and/or gene sequences corresponding to three different categories of type I AFPs have been obtained. These encode either small monomeric peptides (≥37 aa) found in blood (which we will call circulating isoforms) [33], similar peptides lacking secretory signal peptides found in skin and other peripheral tissues (which we will call skin isoforms) [34], and the large, dimeric, highly-active circulating isoform (195 aa/monomer) that remained undiscovered until 2004 [35], [36]. All type I AFPs are alpha-helical, Ala-rich (>50 molar %) and show other similarities in their amino acid compositions. Those from flounder [33], [37] and cunner [32] possess an 11-aa repeat motif of *i*, *i*+4, *i*+8 with Thr at position *i*, which the snailfish sequences lack [38]. Sculpins also produce different categories of AFPs, including a skin isoform from shorthorn sculpin that is longer (92 aa) and lacks the repeats [39] and a skin isoform from longhorn sculpin that is shorter (42 aa) and contains the repeats [25]. The authors also noted that this latter sequence shares a similar N-terminus (MDAPA) and putative salt bridges with several other type I AFPs [25]. In fact, the skin isoforms of three species are so similar that they can be aligned with their 11-aa repeats in register (Fig. 1B).

The similarities between the type I AFPs would suggest that they are all homologous proteins. However, we have carried out more detailed analyses that suggest this is not the case. The additional sequences we have obtained from shorthorn sculpin show that the coding sequences are highly mutable compared to the non-coding sequences. They have also enabled us to compare both the coding and non-coding regions within and between groups of fish. Differences in codon usage, flanking sequences and other properties suggest that the genes from the four different groups of fish are not derived from the same progenitor. We conclude that the similarities between the proteins arose by convergence to a stable α-helical platform with ice-binding capability.

## Materials and Methods

### Collection of Fish and Fish Tissues

This study was carried out in accordance with the guidelines outlined by the Canadian Council for Animal Care. The specific protocols used for the handling of fish under Animal Utilization Protocol number 06-174-F were approved by Memorial University of Newfoundland’s Institutional Animal Care and Use Committee, and were reviewed on an annual basis. Shorthorn sculpin and their egg masses were collected from Conception Bay, Newfoundland, by the Ocean Science Centre (OSC) Field Services crew (SCUBA divers) under a Canada Department of Fisheries and Oceans Experimental License (NL-1587-13). The live fish were brought to the OSC and maintained in aquaria supplied with flowing sea water under ambient conditions. The sculpin were removed from the tank and euthanized by an overdose of MS222 prior to the removal of tissues. The sculpin egg masses were collected by the SCUBA divers brought to the OSC and maintained until they hatched. The larvae were then removed from the tank and euthanized by plunging them into liquid nitrogen.

### Cloning of Shorthorn Sculpin AFP Sequences

All sequences were amplified by PCR using genomic DNA from adult liver or by RT-PCR using RNA from adult liver or whole fry (∼20 mm in length). Two pairs of nested primers were designed from conserved sequences within the 5′ and 3′ untranslated regions of the two known skin sequences from shorthorn sculpin (GenBank #AF305502.1) and longhorn sculpin (AF306348.1). Two additional overlapping primers were designed to span the start codon with differing numbers of degenerate codons corresponding to the amino acids found at the N terminus of shorthorn sculpin isoform SS-8 [26]. The primer sequences are shown in Fig. S1.

Primers were used in all possible combinations with nested primers used in some reamplifications. Primers concentrations were varied according to degeneracy from 0.2 µM for non-degenerate primers to 1 µM for the most degenerate. Reaction conditions were as follows; initial denaturation of 95°C for 5 min followed by 30 cycles (primary amplifications) or 20–25 cycles (reamplification of 1/100^th^ of the prior reaction) of 95°C for 5 min, 50–53°C for 1 min and 72°C for 2 min followed by a final extension of 10 min at 72°C. Taq DNA polymerase was used with 1.5 to 2.5 mM MgCl_2_ and Q-solution as per manufacturer’s instructions (Qiagen, Toronto, Ontario, Canada). PCR products were subcloned using the TOPO TA cloning kit (Invitrogen, Carlsbad, CA) according to manufacturer’s instructions and sequenced (Cortec, Kingston, Ontario, Canada).

### Codon Usage and Other Bioinformatic Analyses

Sequence manipulation, alignments and dot matrices were done using DNAman (Lynnon Corporation, Pointe-Claire, Quebec, Canada). All alignments were manually edited as necessary, with emphasis placed on aligning the infrequent non-Ala codons within the coding regions.

The coding sequences of all known nuclear-encoded genes and cDNAs from each species or species group examined were downloaded from the NCBI non-redundant (nr) database. If two or more sequences from any species shared greater than 65% DNA sequence identity, only the longest was retained. Low quality sequences, judged by the presence of frameshifts or ambiguous bases as well as short sequences (fewer than 50 codons) were also excluded from the winter flounder dataset, but for the others, only the ambiguous codons were removed, as fewer sequences were available. Sequences were trimmed to remove partial codons at either end and the coding sequences were verified using the transeq tool in the EMBOSS suite [40] at the Galaxy website (http://galaxy.tuebingen.mpg.de/). Codon usage was calculated using the cusp tool within the same suite. ESTs were only available for two of the species and were not used for the following reasons. There was a sufficient number of sequences within the nr database for winter flounder, and of the twelve from *Cyclopterus lumpus* (within the snailfish superfamily), two were short, three lacked homologs that could verify potential open reading frames and most of the others were of low quality as they appeared to contain frameshifts.

## Results

### New Shorthorn Sculpin Sequences show Considerable Coding Region Diversity

**Figure 2:**
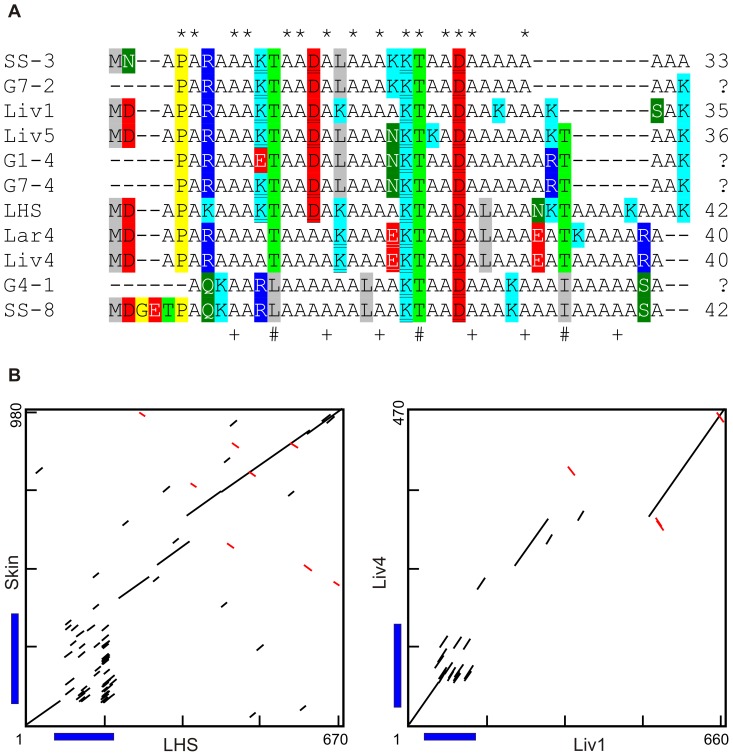
Sculpin AFPs. A) Alignment of Sculpin AFPs. New sequences from shorthorn sculpin cDNAs from liver (Liv), larvae (Lar) or genomic DNA (G) are compared to known shorthorn skin (Skin) and longhorn skin (LHS) sequences. As the deduced peptide sequences are low complexity, they were aligned based on the DNA sequence alignment, which is shown in Fig. S1 along with the accession numbers. Thr is highlighted light green and other polar residues are highlighted dark green with white font. Basic residues are highlighted cyan (Lys) or blue (Arg), acidic are highlighted red in black (Asp) or white (Glu) font, hydrophobic residues (except Ala) are highlighted gray and exceptional residues (Pro and Gly) are highlighted yellow. B) Dot matrix comparisons of selected sculpin isoforms. A line indicates a match of at least 9 out of 10 bases. Coding regions are denoted by blue bars.

Additional AFP sequences encoding variants of the type I AFP from shorthorn sculpin were amplified from both cDNA and genomic templates (Fig. 2A). Eight unique clones are shown, most of which were recovered more than once from different templates and/or by using different primer combinations, and are, therefore, unlikely to contain PCR-generated artefacts. The non-coding regions of these variants showed considerable sequence similarity although insertions and deletions, as well as length heterogeneity in simple sequence repeats, were the norm within the 3′ untranslated regions (3′ UTRs) (Fig. S1 and S2). The four sequences amplified from genomic DNA do not contain any introns.

The deduced AFP sequences from the newly obtained shorthorn sculpin sequences are more similar to the 42-aa longhorn sculpin skin sequence (LHS) [25] (Fig. 2A) than to the previously known 92-aa shorthorn sculpin skin sequence (Skin) [39]. The coding sequences of these two skin isoforms cannot be reliably aligned, as shown by the large number of scattered short diagonals on the dot matrix comparison (Fig. 2B). Most of the length variation in the 33–42 aa isoforms occurs near the C terminus, with gaps up to 9 aa in length (Fig. 2A). The extreme C termini are of two types, one of which resembles that found in LHS (represented by five sequences: G7–2, Liv1, Liv5, G1–4, G7–4) and the second of which is novel (represented by three sequences: Lar4, Liv4, G4–1) (Fig. 2A and S1). Two clones resemble circulating isoforms previously sequenced by Edman degradation (Fig. 2A). The first is G4–1, which matches the updated sequence of SS-8 (see [38] vs. [26]) and the second (G7-2) is similar to SS-3 [26]. These shorter isoforms all show a repeat pattern in which Thr is found at every 11^th^ position, except at two positions in SS-8 where Ile or Leu substitute. Most have two potential helix-stabilizing salt bridges in which three residues separate a basic and acidic residue. Other notable residues include the Asp and Pro near the N-terminus that presumably form a helix-capping structure.

Despite the differences between the deduced AFP sequences of the long and short isoforms and the variability within the short isoforms, the genes encoding these proteins are clearly related, as their UTRs are highly similar. This is shown in representative dot matrix comparisons (Fig. 2B, 90% identity cut off) between the skin isoforms of shorthorn (Skin) and longhorn sculpin (LHS) and between two divergent shorthorn sculpin sequences (Liv1 and Liv4). The 5′ UTRs of all clones are >90% identical and since they share an in-frame stop codon 15 bases upstream of the start codon (Fig. S1), they clearly do not encode secretory signal peptides. The 3′ UTRs are also highly similar, with over 90% identity between overlapping regions as shown by the diagonals in the dot matrices (Fig. 2B), although they do show evidence of at least eight insertion and/or deletion events (Fig. S2). Indeed, the sequence variability in the UTRs is lower than that found within the coding regions, as the latter may be more prone to recombination due to the prevalence of Ala codons. The pattern of insertions and deletions in the 3′ UTRs of LHS and Liv5 (from two different species) are identical (Fig. S2) and distinct from those of Liv4, suggesting that at least some of these sequences duplicated and diverged prior to the divergence of the longhorn and shorthorn sculpins.

### The Protein Sequences of all Type I AFPs Share some Characteristics

The hallmark of type I AFPs is their high content of Ala residues, alpha-helicity and amphipathic character. The type I AFP alpha-helix has a slightly different periodicity (3.7 residues/turn) from the classic alpha-helix (3.6 residues/turn), which causes the intrahelical hydrogen bonds to bifurcate such that the carbonyl groups can hydrogen bond to water [36]. This interaction with the solvent helps keep these relatively hydrophobic helices in solution at the low mM concentrations needed to inhibit ice crystal growth down to and beyond the freezing point of seawater.

**Figure 3:**
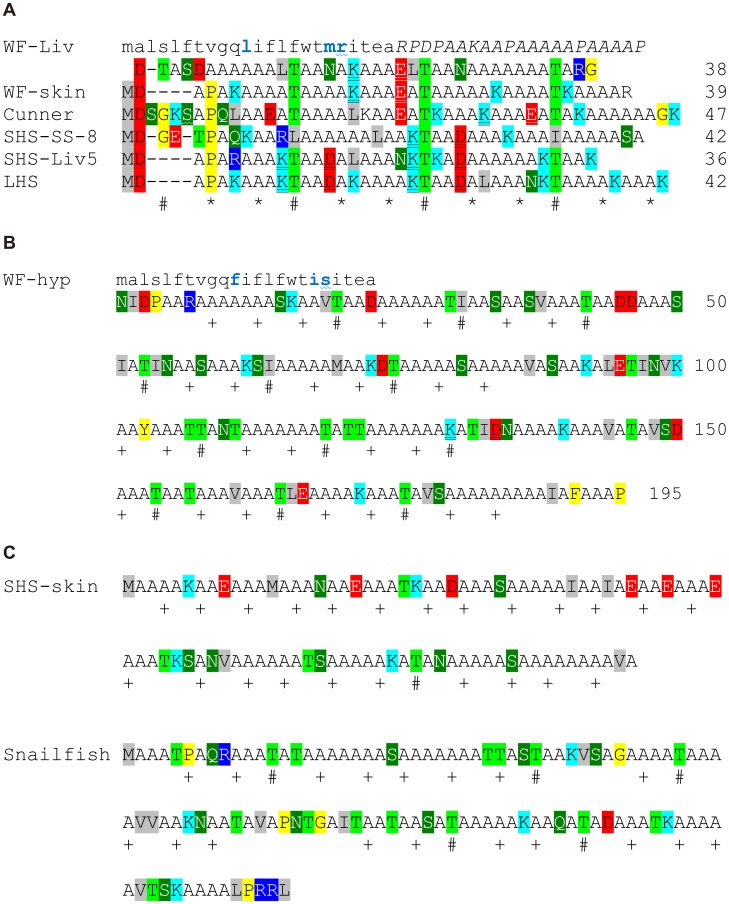
Representative type I AFPs showing their diversity both within species and between species. Symbols and coloring are as in Fig. 2A. A) Alignment of smaller skin and circulatory isoforms from winter flounder liver (WF-Liv, M63478.1) and skin (WF-skin, M63478.1), cunner (JF937681.2), shorthorn sculpin (SHS) SS-8 [38]and Liv5, longhorn sculpin (LHS, AF306348.1) and cunner (JF937681.2). Only WF-Liv possesses a signal peptide (lower case font, difference relative to WF-hyp in blue) and pro-peptide (italics) which is shown on the line above the mature AFP sequence. Amino acids encoded by codons interrupted by an intron in the cunner [71] and flounder liver sequences are indicated with a wavy underline. The intron within the flounder skin gene lies within the 5′ UTR. B) Sequence of the hyperactive type I AFP from winter flounder (WF-hyp, EU188795.1) denoted as in Fig. 2A. This circulating isoform is dimeric and possesses a signal peptide (lower case font) but no pro-sequence. C) Sequence of the two atypical type I AFPs of intermediate length from shorthorn sculpin skin (SHS-skin, AF305502.1) and dusky snailfish (AY455863.1). Thr is seldom found in position *i* of the 11 aa *i*, *i*+4, *i*+8 pattern of ice-binding residues and this pattern is not necessarily continuous in these longer AFPs. Neither AFP possesses a signal peptide or prosequence.

As mentioned in the Introduction, the short skin AFPs of different species are quite similar and share a common 11-aa repeat periodicity, with an *i*, *i*+4, *i*+8 pattern of Thr/Ala/Ala, relative to Thr at position *i*, on the ice-binding surface (Fig. 1B, [41]). This motif is also found in the newly obtained shorthorn sculpin isoforms (Fig. 2A and 3A) as well as the short circulating isoforms of flounders, most of which contain three repeats (Fig. 3A and not shown). These short AFPs typically contain an acidic residue at or near the N-terminus that reinforces the helix dipole, along with a Pro and/or Gly a few residues further along. The most common non-Ala residues besides Thr are charged. There are quite a few basic and acidic residues with optimal separation along the helix (*i*, *i*+4) to form helix-stabilizing salt bridges. Gly and Pro are only found near the termini and aromatic residues are completely absent. On the surface, it would be reasonable to conclude that these short AFPs are homologs. However, the evidence described below suggests that the type I genes from the four different superfamilies of fishes are, in fact, not related.

### Type I AFPs can show Considerable Variability, Even within a Single Species

The longer AFPs are quite distinct from the short ones and fall into two categories. The hyperactive flounder AFP (WF-hyp, Fig. 3B) is approximately five times longer than the short AFPs and is dimeric [36], [37]. It is preceded by a signal peptide highly similar to those found in the conspecific circulating isoforms, but it lacks the prosequence. The 11-aa repeat pattern is present in four sets of three repeats. Notable differences include an increase in polar residues such as Thr at the expense of charged residues and a paucity of acidic and basic residues with an *i*, *i*+4 spacing. So, unlike the monomeric forms that are stabilized by intrachain salt bridges, this dimeric AFP is presumably stabilized through its dimerization interface. The other longer AFPs, from shorthorn sculpin skin (SHS-skin [39]) and dusky snailfish (which is identical to that of Atlantic snailfish isoform except for four out of five residues at the C terminus [42]), are intermediate in length (Fig. 3C) and also appear to lack intrachain salt bridges. They do not show the strict 11-aa spacing between Thr of the others, although the *i*, *i*+4, *i*+8 pattern can be mapped onto the sequences with Thr frequently found at position *i* in the snailfish sequence. The snailfish isoform is unique in that it has several internal Gly and Pro residues that might interrupt helix continuity. Therefore, the only consistent feature shared by all of the type I AFPs is that they are Ala-rich, ranging from 53 mol% in cunner to 71 mol% in the shorthorn sculpin skin isoform.

### The Short Type I AFPs from the Four Different Superfamilies have Unique Features

Despite the similarities between the short isoforms, the positions of the charged residues are not well conserved (Fig. 3A). In addition, the only short sequences to have secretory signal peptides, as well as prosequences, are the liver sequences from the flounder. Furthermore, the known gene structures differ. The sculpin genes do not contain an intron whereas the flounder and cunner sequences do, but the location of these introns are different. The cunner intron interrupts the sequence encoding the mature AFP whereas the flounder intron lies within the region encoding the signal peptide. Both the hyperactive (Fig. 3B) and skin (not shown) flounder genes retain this intron position, but as the skin variant lacks the signal peptide, the homologous first exon is non-coding,

### The Ala Codon Usage is Dramatically Different within the AFP Genes of Three of the Four Species

**Figure 4:**
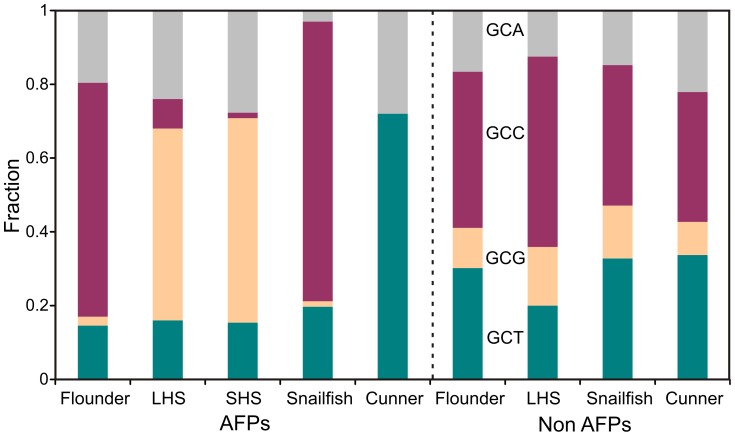
Ala codon usage in type I AFPs. The height of the color bars represents the fraction of each of the Ala codons in each dataset or sequence. The cDNAs used encoded the following AFPs; WF–hyp, LHS, SHS–skin, Atlantic snailfish AFP and cunner AFP. The number of non-AFP sequences used for each group is as follows: winter flounder, 70; longhorn sculpin, 10; cunner, 10 and four species in the snailfish family (Liparidae), 8. The accession numbers for these sequences are listed in Text S1.

The dominance of Ala codons in type I AFPs provides the most statistically significant data about codon usage for these relatively short sequences. A comparison was made using both AFP and non-AFP sequences from all four groups of type I AFP-producing fishes (Fig. 4). There are striking differences between the Ala codon usage in the AFP genes. Sculpin uses primarily GCG (>50%) whereas cunner uses GCT (>70%). Flounder and snailfish show a strong bias towards GCC (63% and 76%, respectively). A comparison of the non-AFPs shows that these biases are not species specific as all of the fish show a slight preference for GCC followed by GCT. The flounder dataset is likely most representative as it is based upon 70 sequences whereas the others are based upon only eight to ten.

A comparison was also made between the codon usages within the different categories of AFP genes from the same species. Reliable alignments cannot be made between either the flounder hyperactive and short sequences or the longhorn and shorthorn sculpin skin sequences (Fig. 3). For the sculpin skin sequences, the two proteins share few common features other than Ala-richness. Nevertheless, for both sculpin sequences, GCG is the preferred codon (52 or 55%) followed by the GCA codon (24 or 28%, Fig. 4). The hyperactive and short (HPLC6) isoforms of winter flounder preferentially use GCC (63 and 71% respectively). Similar patterns of Ala codon usage are also observed between all other isoforms within the same or closely related species (not shown). These codon preferences are not caused by bias in the GC content of the flanking sequences, as the non-coding portions of the cDNAs, as well as the flounder genomic clones, have similar nucleotide contents (Fig. S3). All have a GC content between 37 and 44%. Taken together, the codon usage preferences in the cunner and sculpin AFP sequences, as compared to those of the flounder and snailfish, provide evidence that these genes may have arisen from at least three different progenitors.

### The Non-coding Sequences of the Four Species are Entirely Dissimilar

**Figure 5:**
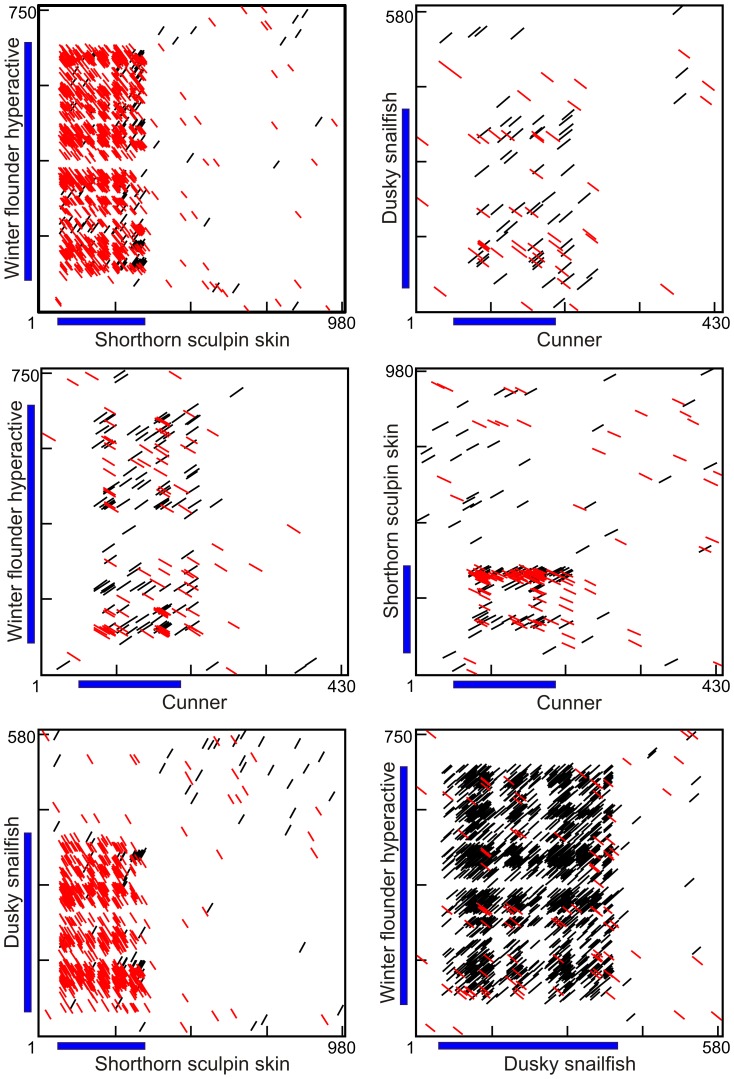
Dot matrix comparisons of type I cDNAs from the four different groups of fishes. A line indicates a match of at least 9 out of 12 bases with red indicating a sense/antisense match. The blue bars denote the coding region (signal peptides excluded). These sequences correspond to those shown in Fig. 3.

Dot matrix comparisons were performed on all possible combinations of representative sequences from the four groups of species (Fig. 5). The longest sequences were selected from species in which multiple sequences were available (shorthorn sculpin and winter flounder) but the results were similar when other isoforms were used (not shown). What is immediately apparent is that there are only sporadic, insignificant short sequence matches in the non-coding regions in all pair-wise comparisons. This is even the case for the two species within the same order (sculpin and snailfish, Fig. 1A). An interesting aside is the numerous scattered matches between the first 30 bp downstream of the cunner coding sequence and the coding regions of the other sequences. This region may contain past coding sequence lost following a single base deletion as it has the potential to encode seven Ala residues.

The lack of similarity between the 5′ and 3′ untranslated regions (UTRs) of the AFPs from these four groups of fishes is not meaningful if there has been sufficient time for these sequences to diverge beyond recognition. Currently, there are too few high-quality sequences containing sufficiently long UTRs to compare non-AFP sequences from the AFP-producing species. Therefore, sequences from related species were used. A total of 14 suitable mRNA sequences are known for the roughskin sculpin, *Trachidermus fasciatus*. As the snailfish superfamily was devoid of suitable sequences, comparisons were made with species in a more divergent scorpaeniform suborder and homologues were identified for six of these genes. Homologues were also identified for at least six genes in fishes from the same suborders as cunner (Labridae) and flounder (Pleuronectoidei). Sequences for three cDNAs (myostatin, peroxiredoxin and β-actin) were available for all four fish groups and pairwise dot matrix comparisons for myostatin, as well as representative examples for some of the other genes, are shown in Fig. S4. In all cases, similarity was found within at least one of the UTRs of these genes. Not surprisingly, it was highest between the sculpin and other scorpaeniforms but was still clearly evident in comparisons with sequences from pleuronectiforms, even though this is the most divergent of the four groups. Therefore, the lack of similarity between any of the UTRs in the type I AFP genes from the four groups would be unexpected had they arisen from a common ancestor.

As all type I AFPs are rich in Ala, it is not surprising that many matches are observed between the coding sequences of the four cDNAs. However, these are short and are scattered throughout the entire coding region such that these sequences cannot be unambiguously aligned. Again there is no evidence of homology. The coding sequences of snailfish and flounder show a density of matches because of their shared preferential use of the GCC codon for Ala (Fig. 5). The antisense coding sequences of both these cDNAs show a forest of matches to the sculpin sequence because antisense runs of GCC codons, frameshifted by one base, are complementary to the runs of GCG codons seen in the sculpin. Cunner, which preferentially uses the GCT codon, shows the fewest matches. This is because none of the other species preferentially use the GCT codon or the GCA codon, runs of which are complementary to the GCT codon.

### There are no Obvious Progenitors for Type I AFPs in the Databases

BLAST and tBLASTn searches were performed on teleost fish sequences found in all relevant GenBank databases (nr, EST, GSS etc.) [43] using either the non-coding or coding sequences of type I AFP genes or the protein sequences. There were no matches to the non-coding sequences that were strong enough or extensive enough to indicate homology.

We repeated the BLAST searches using the AFP coding regions and protein sequences with the low complexity filters turned off. Thousands of hits were obtained from both genomic and EST databases (not shown). Most of the hits to genomic DNAs were to microsatellites containing trinucleotide repeats (GCT or GCA) that could potentially encode runs of Ala residues. The alternative runs (GCC or GCG) were nowhere near as frequent and were found mostly in AFPs and in coding sequences containing runs of Gly residues such as the keratin and eggshell proteins as found by Evans and Fletcher is 2005 [44]. These authors pointed out that a frame shift could convert the Gly-rich regions of these proteins to Ala-rich regions. Coding sequences rich in other amino acids could also generate Ala-rich sequences if frameshifted or read on the opposite strand (Table 1). The only AFPs with a codon preference consistent with a sense frameshift on a Gly-rich protein are those from sculpins. Flounder and snailfish are consistent with the antisense strand, and cunner is consistent with neither. Alternatively, the genes could have arisen following duplication of a short segment of DNA encoding Ala residues with one particular codon. This would be analogous to the mechanism by which a 9 bp segment of a trypsinogen gene was duplicated many times to generate the AFGP [19]. Further expansions and contractions could then be mediated by the similarities within either the Ala-coding stretches or the 11 aa repeat. However, until the progenitors of type I AFPs are discovered, the mechanism of origin of these Ala-rich proteins cannot be ascertained.

**Table 1:** Amino acids encoded in alternative reading frames by adjacent Ala codons.

Species or group	Preferred codon	Alternative sense	Alternative antisense
Cunner	GCT	Leu/Cys	Ser/Ala/Gln
Flounders/snailfishes	GCC	Pro/Arg	Gly/Ala/Arg
Sculpins	GCG	Arg/Gly	Arg/Ala/Pro

## Discussion

The estimated times of divergence [45]–[47] of most of the different groups of fishes that produce AFPs lie within the time frame when the earth was quite warm and largely devoid of ice [28]. Some fishes may have produced AFPs prior to this warm period, but it is doubtful they would have retained that capability throughout the ∼200 Ma period between glacial epochs. This climate history could explain why distinct AFP types are found in different fishes (Fig. 1A), but not why different fishes produce similar AFPs. In the case of the globular type II AFPs, lateral gene transfer has been proposed as the mechanism behind the >90% identity within the intronic sequences of the genes from the very divergent smelt and herring species [22], [48], [49]. A different, but equally compelling explanation has been advanced for the AFGPs found in cods and notothenioids. These proteins, composed primarily of simple tripeptide repeat (AAT or PAT), likely arose by convergent evolution [20]. The type I AFPs are somewhat more complex than the AFGPs as their repeat, if present, is longer (11 aa) and more variable (Fig. 1B). The recent discovery of new type I AFP sequence from cunner [32], as well as the additional sculpin sequences obtained in this study, has enabled us to carry out detailed comparisons of these sequences from the four different groups of fish (Fig. 1A). We propose that they arose by convergent evolution in all four lineages.

We have expanded the repertoire of shorthorn sculpin AFP sequences from one to nine and have confirmed the sequence of SS-8 that was previously determined by Edman degradation [26], [38]. All of the encoded peptides are similar to traditional type I sequences, such as the skin isoform known from longhorn sculpin [25], as they are short (33 to 42 aa) and display the 11-aa repeat pattern. Some isoforms were more similar to the longhorn sculpin skin isoform than to other conspecific isoforms, which was particularly evident from the patterns of insertions and deletions in the 3′ UTRs. This would suggest that some of these genes have been undergoing divergent evolution prior to the divergence of the two sculpins lineages. The similarity between the UTRs of the short isoforms and long isoform indicates that these genes are derived from a common precursor and that the coding sequences are highly mutable, likely by expansion and contraction through recombination at Ala-coding stretches. This phenomenon was also observed in the three forms of AFP from winter flounder (skin, circulating short and circulating hyperactive) which showed even greater plasticity in their coding regions [37]. They are also expressed differentially or in different tissues [34], [50] indicating regulatory and functional divergence following multiple rounds of duplication.

When all type I AFPs are compared, their primary similarity is that they are Ala-rich. This exceptional bias in amino acid composition would provide greater than the 25% sequence identity for any type I AFP pair comparison. The highest sequence similarity is amongst the shortest isoforms (Fig. 1B) [25], [32], [34]. Structures for two of these small isoforms have been determined to be single, stand-alone alpha-helices [51], [52]. As Ala is the amino acid with the highest propensity to form α-helices [53], it is not surprising that this residue dominates in type I AFPs. Acidic residues at the N terminus and basic residues at the C terminus are involved in capping structures [52] and in counteracting and interacting with the dipole moment inherent in all α-helices [54]. Acidic and basic residues separated by three residues have been shown to form helix-stabilizing salt bridges [55] and these must not reside upon the ice-binding surface. Additionally, as all of the side chains in these isolated helices are surface exposed, large hydrophobic residues would be expected to decrease solubility. Given these constraints, it is not surprising that the composition of all type I AFPs is quite similar and that the short isoforms share superficial sequence similarity.

An additional feature shared by some type I AFPs is an 11-aa periodicity involving Thr residues. Thr is an important ice-binding residue in the AFPs of some other organisms. In fact, these other AFPs also demonstrate convergent evolution. Those from mealworm beetles [56] and the spruce budworm moth [57], [58] consist of repeats of a different lengths (12 or 19 residues), have completely different disulfide-bonding patterns, and coil with opposite handedness. Yet both have flat ice-binding surfaces consisting of a double row of sterically aligned Thr residues (TxT motif) that reside upon one face of a β-helical platform. The AFP from the bacterium, *Marinomonas primoryensis*, is also β-helical, but here the ice-binding face lies upon a turn of the β-helix and consists of TxN rather than TxT motifs [59]. Thr is also found on the ice-binding surfaces of β-helical AFPs from grass [60] and fungi [61] albeit with lower frequency and regularity. Therefore, it is not surprising that most type I AFPs also contain regularly-spaced Thr residues. Here the 11-aa periodicity places them on one side of the helix and their sidechains are also sterically constrained [52]. As so many of the residues in these small proteins are functionally important, it is entirely feasible that the skin AFPs of sculpins, cunner and flounders are similar due to convergent rather than divergent evolution. What is apparent from Fig. 1B is that the Ala and Thr residues that have been determined to be important for binding to ice [41], [62] are shared between the AFPs of the three species but the non-Ala residues at the other positions are actually quite variable, as indeed they are when comparing isoform sequences from winter flounder and closely related righteye flounders. Therefore, there is no evidence for homology based upon residues that are less functionally constrained.

An emerging mechanism to explain AFP binding to ice is the ‘anchored clathrate water’ hypothesis [59]. In this process, regularly spaced hydrophobic groups will be surrounded by water ‘cages’ or clathrates. When some of these clathrate waters are hydrogen bonded to nearby hydrophilic groups the whole water network becomes more ordered and ice-like, ready to merge with the quasi-liquid water at the surface of ice. Thr is an ideal ice-binding residue because it has a methyl group adjacent to a hydroxyl group. Ala has also been demonstrated to be an important ice-binding residue in type I AFPs [38], [41]. It has a methyl group as its side chain which is small and can allow some access to the backbone peptide bond for hydrogen bonding anchoring. This is particularly true with the type I AFP helix where the intra-helical hydrogen bonds are bifurcated and project the carbonyl groups towards the solvent [36]. Not all AFPs employ Thr as an ice-binding residue, as demonstrated by the poly-proline II type snow flea AFP isoforms, in which the ice-binding face is dominated by Ala residues [63], [64]. Therefore, the lack of Thr in favor of Ala on the longer type I skin isoforms, from shorthorn sculpin [39] and the snailfishes [42] is consistent with other know ice-binding surfaces. Therefore, we propose that type I AFPs have arisen independently on α-helical platforms and have favoured Ala as both a structural and ice-binding residue.

The similarities and differences between the type I AFP families found in the four groups of fishes are summarized in Table 2. For example, secretory signal peptides are only found in flounder even though AFPs are found in the blood of fishes from the other three groups. The skin isoforms of flounder are distinctly different from the circulating isoforms [34], whereas they are the same in snailfish [42]. The structural characteristics of some of the longer isoforms likely vary more dramatically than those of the shorter isoforms as, for example, the snailfish AFPs possess the highest density of internal helix-disrupting Pro or Gly residues, and the large flounder isoform is dimeric. Perhaps most telling are the dramatic differences between the nucleotide sequences. The lack of an intron in the sculpin genes, relative to the flounder and cunner genes (which possess introns in different locations), strongly suggests that these genes are not related. Comparisons between the two pufferfish genomes (*Takiugu rubripes* and *Tetraodon nigroviridis*), which diverged ∼32 Ma, as well as human and mouse with diverged ∼61 Ma, indicated that fewer than 0.05% of the introns were lost in each lineage and none were gained [65]. The intron differences in the AFP genes would be unlikely to occur in the ∼125 Ma or less since these fish groups diverged. As well, the UTRs lack any significant similarity. Furthermore, the Ala codon usage differs. Of the four Ala codons, GCC is strongly preferred in flounder and snailfish, GCT is preferred in cunner and GCG in sculpins (GCG), whereas all groups favor GCC in their other genes. Gly-rich keratin sequences (which could encode Ala (GCG) by frameshifting or on the antisense strand (GCC)) or chorion sequences containing an Ala-rich region were obtained during library screening for snailfish AFP sequences. This led to the hypothesis that one of these may have been the precursor of the snailfish AFP gene [44]. Similar scenarios could explain any codon bias as, for example, a frameshift on a Pro-rich sequence could give rise to GCC codons whereas a Leu-rich sequence could give rise to GCT codons. There are also repetitive non-coding sequences that could give rise to Ala-rich peptides if they were co-opted into a coding sequence. As the coding sequences of the AFPs are highly mutable, presumably due to unequal recombination in runs of Ala codons, the progenitor genes are unlikely to be identified through codon sequence alone. Unfortunately, the progenitors remain unknown, as the coding and flanking sequences of the AFP genes are not similar enough to the keratin or chorion genes, or to any other non-AFP sequences in the databases, to deduce homology. What these searches do indicate is that there is no shortage of pre-existing repetitive sequences in fish genomes that could be co-opted to produce Ala-rich peptides that could eventually evolve into fully-functional AFPs. Taken together, this strongly suggests that the type I AFP family is not a true family at all but is convergence of α-helical progenitors towards the optimal composition for stability and ice binding.

**Table 2:** Properties of the type I AFPs from the four groups of fish.

Species or group	Different categories	Size range (kDa)	Skin/blood isoforms different	Signal peptides	Pro-peptide	Thr spacing	Codon preference	Internal Pro/Gly	Introns
Cunner	2^a^	4–7	Yes	No	No	Yes/?	GCT	No/?	1
Snailfishes	1	9–10	No	No	No	No	GCC	Yes	?
Flounders	3	3–33	Yes	Yes/No	Yes/No	Yes	GCC	No	1
Sculpins	2	3–8	Yes/No^b^	No	No	Yes/No	GCG	No	0

a The sequence of a larger circulating isoform is unknown but an accurate mass and rough composition were determined from an enriched sample [66].

b Longhorn sculpin skin and circulating isoforms are highly similar, but one shorthorn sculpin cDNA from skin encodes a distinct isoform.

## Supporting Information

Figure S1 **Alignment of shorthorn sculpin cDNA and gene sequences encoding AFPs.** Sequences from shorthorn larval (Lar) or adult liver (Liv) cDNAs or genomic DNA (G) are compared to shorthorn skin (skin) and longhorn sculpin (LHS) sequences. Conserved nucleotides are white with black highlighting and coding sequences are in uppercase font. Note that the coding sequence of the skin isoform cannot be reliably aligned with the other coding sequences. The sequences of the six PCR primers are given below their annealing locations with the two codon insertion in Deg2 underlined. Sequences have been deposited in GenBank with the following accession numbers: G7-2, KF381189; Liv1, KF381183; Liv5, KF381185; G1-4, KF381187; G7-4, KF381190; Lar4, KF381186; Liv4, KF381184; G4-1, KF381188.
(PDF)

Figure S2 **Comparative schematic of sculpin type I AFP genes.** A total of eight new sequences are shown after those with three or fewer silent or non-coding mutations were exuded. They were obtained from cDNAs isolated from liver (Liv) or larvae (Lar) or from genomic DNA (G) and are compared to known shorthorn skin (Skin) and longhorn skin (LHS) sequences. Coding sequences are shown by thick bars, with color gradation approximating relative similarity. Gaps are indicated by hollow thin bars and non-coding sequence by filled bars of intermediate thickness. Gray bars indicate a hypervariable region, containing GT_n_ and G_n_ repeats, from which deletions and fine detail has been omitted. Red bars indicate unique sequence present in but a single clone. Identical symbols (red asterisks or black circles) indicate shared breakpoints.
(PDF)

Figure S3 **Nucleotide content of flanking non-coding regions of selected type I AFP cDNA sequences.** The sequences used are as follows; shorthorn sculpin (skin, AF305502.1), cunner (JF937681.2) and snailfish (AY455863.1). The HPLC6 gene of winter flounder (M62415.1) is also shown in which the UTRs (WF) are scored separately from the intronic, upstream and downstream sequence (WFgene). The total number of nucleotides scored for each is indicated in the gray bar.
(PDF)

Figure S4 **Dot matrix comparisons of non-AFP cDNAs from the four different groups of fishes.** A line indicates a match of at least 9 out of 12 bases. Antisense matches were excluded for clarity. The thin blue bars denote the coding region with thicker hatched yellow bars denoting the untranslated regions. Homologs to 10 of 14 protein-coding non-mitochondrial cDNAs from the roughskin sculpin, *Trachidermus fasciatus*, were found in the non-redundant (nr) database within one or more of the type I AFP producing fish groups (Fig. 1); Labridae (cunner), Pleuronectiformes (flounder) and suborders of Scorpaeniformes external to both sculpins and snailfish. Homologues to three cDNAs were found in all groups and pairwise comparisons are shown for myostatin, with a single comparison to the most divergent group (Pleuronectiformes) shown for peroxiredoxin and β-actin. Four additional comparisons, selected from the seven other groups of homologs, are also shown. The most divergent UTRs (IRAK-4) can be contrasted with the least convergent (β-actin). The GenBank accession numbers for the sequences compared (those used in the figure are bolded, with the gene name as it appears in the figure in italics) are as follows; *myostatin* **GU198192.1** (sculpin), **DQ423474.1** (scorpaeniform), **XM_003458832.2** (labrid), **EU443627.1** (pleuronectiform); *β-actin* HM449124.1 (sculpin), JN226153.1 (scorpaeniform), **XM_005743477.1** (labrid) **HQ386788.1** (pleuronectiform); *peroxiredoxin*-1 **JQ911738.1** (sculpin), AB490894.1 (scorpaeniform), XM_003453360.2 (labrid), **DQ009987.1** (pleuronectiform); galactoside-binding *lectin* JX908825.1 (sculpin), **BT082644.1** (scorpaeniform), **DQ993254.1** (pleuronectiform); *myogenin* **JQ905626.1** (sculpin), XM_005739439.1 (labrid), **EF144128.1** (pleuronectiform); interleukin 1-β (*IL1B*) **JQ319051.1** (sculpin), AB491084.1 (scorpaeniform), **FJ769829.1** (pleuronectiform); interleukin-1 receptor-associated kinase 4 (*IRAK-4*) **JQ319050.1** (sculpin), **XM_003443911.2** (labrid), FJ825148.1 (pleuronectiform); thioredoxin domain containing 17 JQ911737.1 (sculpin), BT083140.1 (scorpaeniform); transferrin JN601701.1 (sculpin), XM_005721327.1 (labrid); myogenic factor JQ905628.1 (sculpin), XM_005740857.1 (labrid).
(PDF)

Text S1 **Accession numbers of coding sequences used to calculate codon usage frequencies.** The non-AFPs are listed first with the total number of sequences used indicated. The accession numbers of the AFPs are listed last.
(PDF)
