# Supplementary material for: Helical Antifreeze Proteins Have Independently Evolved in Fishes on Four Occasions
Source: PLoS One. 2013 Dec 6;8(12):e81285. doi: 10.1371/journal.pone.0081285 (PMC3855684; doi:10.1371/journal.pone.0081285)
Supplement: Figure S1 — Alignment of shorthorn sculpin cDNA and gene sequences encoding AFPs. Sequences from shorthorn larval (Lar) or adult liver (Liv) cDNAs or genomic DNA (G) are compared to shorthorn skin (skin) and longhorn sculpin (LHS) sequences. Conserved nucleotides are white with black highlighting and coding sequences are in uppercase font. Note that the coding sequence of the skin isoform cannot be reliably aligned with the other coding sequences. The sequences of the six PCR primers are given below their annealing locations with the two codon insertion in Deg2 underlined. Sequences have been deposited in GenBank with the following accession numbers: G7-2, KF381189; Liv1, KF381183; Liv5, KF381185; G1-4, KF381187; G7-4, KF381190; Lar4, KF381186; Liv4, KF381184; G4-1, KF381188. (PDF) [file pone.0081285.s001.pdf]

```
G7-2      1 ------------------------------------------------------------
Liv1      1 ------------------------------------------------------------
Liv5      1 ------------------------------------------------------------
G1-4      1 ------------------------------------------------------------
G7-4      1 ------------------------------------------------------------
LHS       1 ggcatcttcttctaacaccctggaagagacagacctcgagtttatcatcatcctttattg
Lar4      1 ------------------------------------------------------------
Liv4      1 ------------------------------------------------------------
G4-1      1 ------------------------------------------------------------
Skin      1 ------------------------------------------------------------

G7-2      1 ------------------------------------------------------------
Liv1      1 --------------------------------------------tttccaaacgcaccga
Liv5      1 --------------------------------------------tttccaaacgcaccga
G1-4      1 ------------------------------------------------------------
G7-4      1 ------------------------------------------------------------
LHS      61 agagaaacagttgtgactcatcacgaagttgttgatctttctcttttccgaacgcaccga
Lar4      1 --------------------------------------------tttccaaacgcaccga
Liv4      1 --------------------------------------------tttccaaacgcaccga
G4-1      1 ------------------------------------------------------------
Skin      1 ------------gtgactcatcaggaagttgttgatctttctctgttccaaacgcaccga
                   5′1 GGAAGTTGTTGATCTTTCTCT      5′2 CACCGA

G7-2      1 -------------------------ACCAGCAAGAGCAGCGGCAAAGACGGCAGCAGACG
Liv1     17 gctaaacaaaagtgagaATGGACGCACCAGCAAGAGCAGCGGCAAAGACGGCGGCGGACG
Liv5     17 gataaacaaaagtgagaATGGATGCACCAGCAAGAGCAGCGGCAAAGACGGCAGCAGACG
G1-4      1 -------------------------ACCAGCAAGAGCAGCGGCAGAGACGGCAGCAGACG
G7-4      1 -------------------------ACCAGCAAGAGCAGCGGCAAAGACGGCAGCAGACG
LHS     121 gctaaacaaaagtgagaATGGACGCACCAGCAAAAGCAGCGGCAAAGACGGCAGCGGACG
Lar4     17 gctaaacaaaagtgagaATGGACGCACCAGCGAGAGCAGCGGCAGCGACGGCGGCGGCAG
Liv4     17 gataaacaaaagtgagaATGGACGCACCAGCAAGAGCAGCGGCAGCGACGGCGGCGGCGG
G4-1      1 -----------------------------AGCGCAGAAAGCGGCAAGGCTTGCGGCGGCGG
Skin     49 gctaaacaaaagtgagaATGGCGGCGGCGGCGAAGGCGGCGGAGGCGGCGGCAATGGCGG
            GCTAAACAAAAGTG
            Deg1 ACAAAAGTGAGAATGGAYGG
                    Deg2 GAGAATGGAYGGNGARACNCC

G7-2     36 CGTTGGCTGCGGCGAAGAAGACGGCGGCGGACGCTGCGGCTGCGGCG-------------
Liv1     77 CGAAGGCTGCGGCGGCCAAGACGGCGGCGGACGCTGCTAAGGCGGCGGCCAAG-------
Liv5     77 CGTTGGCTGCGGCGAACAAGACGAAGGCGGACGCTGCTGCGGCGGCGGCCAAGACG----
G1-4     36 CATTGGCTGCGGCGAACAAGACGGCGGCGGACGCTGCTGCGGCGGCGGCCAGGACG----
G7-4     36 CGTTGGCTGCGGCGAACAAGACGGCGGCGGACGCTGCTGCGGCGGCGGCCAGGACG----
LHS     181 CGAAGGCTGCGGCGGCCAAGACGGCAGCGGACGCGTTGGCTGCGGCGAACAAGACGGCGG
Lar4     77 CGAAGGCTGCGGCTGAGAAGACGGCGGCGGACGCTTTGGCGGCGGCGGAGGCTACGAAGG
Liv4     77 CGAAGGCTGCGGCAGAGAAGACGGCAGCGGACGCCTTGGCGGCGGCGGAGGCGACTGCGG
G4-1     33 CGGCGGCTTTGGCAGCGAAGACGGCGGCGGACGCTGCGGCGAAGGCGGCGGCGATTGCGG
Skin    109 CGGCAAATGCGGCGGAGGCGGCGGCAACGAAGGCGGCTGATGCGGCTGCGTCGGCGGCAG

G7-2     84 --------------GCGGCTAAATAAttgg------------------------------
Liv1    130 --------------TCTGCTAAATAAttgt------------------------------
Liv5    133 --------------GCTGCTAAATAAttgg------------------------------
G1-4     92 --------------GCTGCTAAATAAttag------------------------------
G7-4     92 --------------GCTGCTAAATAAttag------------------------------
LHS     241 CGGCGGCCAAGGCGGCTGCTAAATAAttgg------------------------------
Lar4    137 CAGCAGCTGCTAGAGCTTAG----------------------------------------
Liv4    137 CAGCAGCTGCTAGAGCTTAG----------------------------------------
G4-1     93 CAGCAGCTGCTAGTGCTTAG----------------------------------------
Skin    169 CTGCGGCTATTGCGGCTATTGCGGAGGCGGCGGAGGCGGCGGAGGCAGCGGCAACGAAGT
```

```
G7-2     99 ------------------------------------------------------------
Liv1    146 ------------------------------------------------------------
Liv5    149 ------------------------------------------------------------
G1-4    108 ------------------------------------------------------------
G7-4    108 ------------------------------------------------------------
LHS     271 ------------------------------------------------------------
Lar4    157 ------------------------------------------------------------
Liv4    157 ------------------------------------------------------------
G4-1    113 ------------------------------------------------------------
Skin    229 CGGCTAATGTAGCGGCGGCGGCGGCAGCGACGTCGGCCGCGGCAGCAGCGAAGGCTACGG

G7-2     99 ------------------------------------------------------------
Liv1    146 ------------------------------------------------------------
Liv5    149 ------------------------------------------------------------
G1-4    108 ------------------------------------------------------------
G7-4    108 ------------------------------------------------------------
LHS     271 ------------------------------------------------------------
Lar4    157 ------------------------------------------------------------
Liv4    157 ------------------------------------------------------------
G4-1    113 ------------------------------------------------------------
Skin    289 CTAATGCGGCAGCGGCAGCATCAGCAGCTGCAGCAGCTGCAGCAGCAGTTGCGTAGcagt

G7-2     99 ----------------------------------------------caaacatagttaat
Liv1    146 ----------------------------------------------caaacatagttaat
Liv5    149 ----------------------------------------------caaacatagttaat
G1-4    108 ----------------------------------------------caaacatagttaat
G7-4    108 ----------------------------------------------caaacatagttaat
LHS     271 ----------------------------------------------caaacatagttaat
Lar4    157 -------tagagcagttgcctgcttataatgct---------------------------
Liv4    157 -tctccgtagagcagttgcctgcttataatgctcgatatgtggcaacaaacatagttaat
G4-1    113 -tctccttagagcatttgcctgcttataatgctcgatatgtggaaacaaacatagttaat
Skin    349 gtctccgtagagcagttggctgcttataatgctcgatatgtggcaacaaacatagttaat

G7-2    113 ttgttcagtaatgcataaattacactataaggttcttttagggtgtgtgtgcgtgtgtgt
Liv1    160 ttgttcagtaatgcataaattacactataaggttcttttagggtgggtgtgta-------
Liv5    163 ttgttcagtaatgcataaattacactataaggttcttttagggtgtgtgtgtgtgtgtgt
G1-4    122 ttgttcagtaatgcataaattacactataaggttcttttagggtgtgtgt----------
G7-4    122 ttgttcagtaatgcataaattacactataaggttcttttagggtgtgtgt----------
LHS     285 ttgttcagtaatgcataaattacactataaggttcttttagggtgtgtgtgtgta-----
Lar4    183 ------------------------------------------------------------
Liv4    215 ----------------------------------agttaaggatgt--------------
G4-1    172 ttgtgcagtaatgcataagttacactataaggttcttttagggtgtgtgt----------
Skin    409 ttgttcagtaatgcacaagttacactataaggttcttttagggtgt--------------

G7-2    173 gtgt----------gggggggggggg----------------------------------
Liv1    213 --------------gggggtggggg-----------------------------------
Liv5    223 gtgtgtgtgtgtgtgggggggggg------------------------------------
G1-4    172 --------------gggggg----------------------------------------
G7-4    172 --------------gggggg----------------------------------------
LHS     340 --------------gggggggtggggg---------------------------------
Lar4    183 --------------ggggggggggg--tagtatttgattattcgatttgttaa-------
Liv4    228 --------------ggggg-----------------------------------------
G4-1    222 --------------ggggggggggg-----tatttgattattcgatttgttaa-------
Skin    455 --------------ggg----------tagttggctgctctttgatttatgaatgacaaa
```

```
G7-2   189 ---------tatgatttgtccctgcttaattcttaatccct-------------------
Liv1   224 ---------tatggtttgtctatgcttaattcttaatccctgatgttgctgacccaactc
Liv5   247 ---------tatggtttgtctatgcttaattcttaatccctgatgttgctgacccaactc
G1-4   178 ---------tatgatttgtccctgcttaatccttaatccct-------------------
G7-4   178 ---------tatgatttgtccctgcttaattcttaatccct-------------------
LHS    353 ---------tatggtttgtctatgcttaattcttaatccctgatgttgctgacccaactc
Lar4   218 ------------------------------------------------------------
Liv4   233 -----attttatggtttgtctatgcttaattcttaatccctgatgttactgacccaactc
G4-1   254 ------------------------------------------------------------
Skin   491 ttgggattttatggtttgtccatgcttaattcttaatccctgatgttgctgacccaactc
                                                3'2 CTACAACGACTGGGTTGAG

G7-2       ------------------------------------------------------------
Liv1   275 agtgtggtgcttgtttatgttgctgaatatcacaggttattaatacgtaaattcaagtat
Liv5   298 agtgtggtgcttg-----------------------------------------------
G1-4       ------------------------------------------------------------
G7-4       ------------------------------------------------------------
LHS    404 agtgtggtgcttg-----------------------------------------------
Lar4   218 ------------------------------------------------------------
Liv4   288 agtgtggtgcttttttttttttg--------------------------------------
G4-1   254 ------------------------------------------------------------
Skin   551 agtgtggtgcttgtttatgttgctgaatatcacaggttattaatacgtaaattcaagtat

G7-2       ------------------------------------------------------------
Liv1   335 ggaacacacatttattttgttgttgtgagaaatgtattgtgcctatatactcggtactc
Liv5   311 ------------------------------------------------------------
G1-4       ------------------------------------------------------------
G7-4       ------------------------------------------------------------
LHS    417 ------------------------------------------------------------
Lar4   218 ------------------------------------------------------------
Liv4   310 ------------------------------------------------------------
G4-1   254 ------------------------------------------------------------
Skin   611 ggaacacacatgttattttgttgttgtg--------------------------------

G7-2       ------------------------------------------------------------
Liv1   395 aaggacacaaccatcaggatgcaactcgacaactttaataatacttcttctcacacgtat
Liv5   311 ------------------------------------------------------------
G1-4       ------------------------------------------------------------
G7-4       ------------------------------------------------------------
LHS    417 ------------------------------------------------------------
Lar4   218 ------------------------------------------------------------
Liv4   310 ------------------------------------------------------------
G4-1   254 ------------------------------------------------------------
Skin   639 ------------------------------------------------------------

G7-2       ------------------------------------------------------------
Liv1   455 atcacaacatcatatatcagtgcgccctcaactggccatagccggccactacaagaaaca
Liv5   311 ------------------------------------------------------------
G1-4       ------------------------------------------------------------
G7-4       ------------------------------------------------------------
LHS    417 ------------------------------------------------------------
Lar4   218 ------------------------------------------------------------
Liv4   310 -----------------------------------------------------agaaaca
G4-1   254 ------------------------------------------------------------
Skin   639 -----------------------------------------------------agaaaca
```

```
G7-2      ------------------------------------------------------------
Liv1  515 catgcaatcaaacaaaaagcatcttgagacgctcctgttgtgaatcagtttaatcaattt
Liv5  311 -------tcaaacatatagcatcttgagacgctcctgttgtgaatcagtttaatcaattt
G1-4      ------------------------------------------------------------
G7-4      ------------------------------------------------------------
LHS   417 -------tcaaacaaaaagcatcttgagacgctcctgttgtgaatcagtttaatcaattt
Lar4  218 -------tcaaacaaaaagcatcttgagacgctcctgttgtgaatcagcatcatcaattt
Liv4  317 catgcaatcaaacaaaaagcatcttgagacgctcctgttgtgaatcagtttaatcaattt
G4-1  254 -------tcaaacaaaaagcatcttgatacgctcctgttgtgaatcagtataatcaattt
Skin  646 cattcaatcaaacaaaaagcatcttgagacgctcctgttgtgaatcagtataatcaattt

G7-2      ------------------------------------------------------------
Liv1  575 aaatgtgtggttaaaaacccgctgcttagatctcataacaaagaaatgtttttacagccc
Liv5  364 aaatgtgtggttaaaaacccgctgcttagatctcataaccaaaaaatgtttttacagccc
G1-4      ------------------------------------------------------------
G7-4      ------------------------------------------------------------
LHS   470 aaatgtgtggttaaaaacccgctgcttagatctcataaccaagaaatgtttttacagccc
Lar4  273 aaatgtgtggttaaaaacccgctgcttagatctcataaccaagaaatgtttttacagccc
Liv4  377 aaatgtgtggttaaaaacccgctgcctagatctcataaccaagacatgtttttacagccc
G4-1  309 aaatgtgtggttaaaaacctgctgcttagatctcataaccaagaaatgtttttacagccc
Skin  706 aaatgtgtggttaaaaactcactgcttaaatctcataaccaagaaatgcttttacagccc

G7-2      ------------------------------------------------------------
Liv1  635 gggaaaagtgaacctccacaaagatctttcttta--------------------------
Liv5  424 gggaaaagtgaacctccacaaagatctttcttta--------------------------
G1-4      ------------------------------------------------------------
G7-4      ------------------------------------------------------------
LHS   530 gggaaaagtgaacctccacaaagatctttctttagagctaatgtagtagcttaaagatct
Lar4  333 gggaaaagtgaacctccacaaagatctttcttta--------------------------
Liv4  437 gggaaaagtgaacctccacaaagatctttcttta--------------------------
G4-1  369 gggaaaagtggacctccacaaagatctttctttg--------------------------
Skin  766 gggaaaagtgaacctccacaaagatctttctttagagctaatgtagtagcttaaagatct
                                            CTCGATTACATCATCGAATTTC 3’1

G7-2      ------------------------------------------------------------
Liv1      ------------------------------------------------------------
Liv5      ------------------------------------------------------------
G1-4      ------------------------------------------------------------
G7-4      ------------------------------------------------------------
LHS   590 catcagtgttgatgagcaaagtcgttggtgaacaaactgtacctctttaaaaactttttg
Lar4      ------------------------------------------------------------
Liv4      ------------------------------------------------------------
G4-1      ------------------------------------------------------------
Skin  826 catcagtgttgatgagcaaagtcgttggtgaacaaactgtacctctttaaaaacgttttg

G7-2      ------------------------------------------------------------
Liv1      ------------------------------------------------------------
Liv5      ------------------------------------------------------------
G1-4      ------------------------------------------------------------
G7-4      ------------------------------------------------------------
LHS   650 aaggtcagaccggagctaaaactcccaatttatctccatctggttattgagaaatgttgt
Lar4      ------------------------------------------------------------
Liv4      ------------------------------------------------------------
G4-1      ------------------------------------------------------------
Skin  886 aagttcagaccggagctaaaactccc-atttatctccatctggttattgagaaatgttgt
```

```
G7-2          ---------------------------------------------------
Liv1          ---------------------------------------------------
Liv5          ---------------------------------------------------
G1-4          ---------------------------------------------------
G7-4          ---------------------------------------------------
LHS      710  gatttgggtgatttggatgaattaaaagaacctctataaacaaaaaaaaaa
Lar4          ---------------------------------------------------
Liv4          ---------------------------------------------------
G4-1          ---------------------------------------------------
Skin     945  gatttgggtgatttgggtgaattaaaagaacctctataaacaaaaaaaaaa
```
