# Supplementary figures and images for: Helical Antifreeze Proteins Have Independently Evolved in Fishes on Four Occasions
Source: PLoS One. 2013 Dec 6;8(12):e81285. doi: 10.1371/journal.pone.0081285 (PMC3855684; doi:10.1371/journal.pone.0081285)

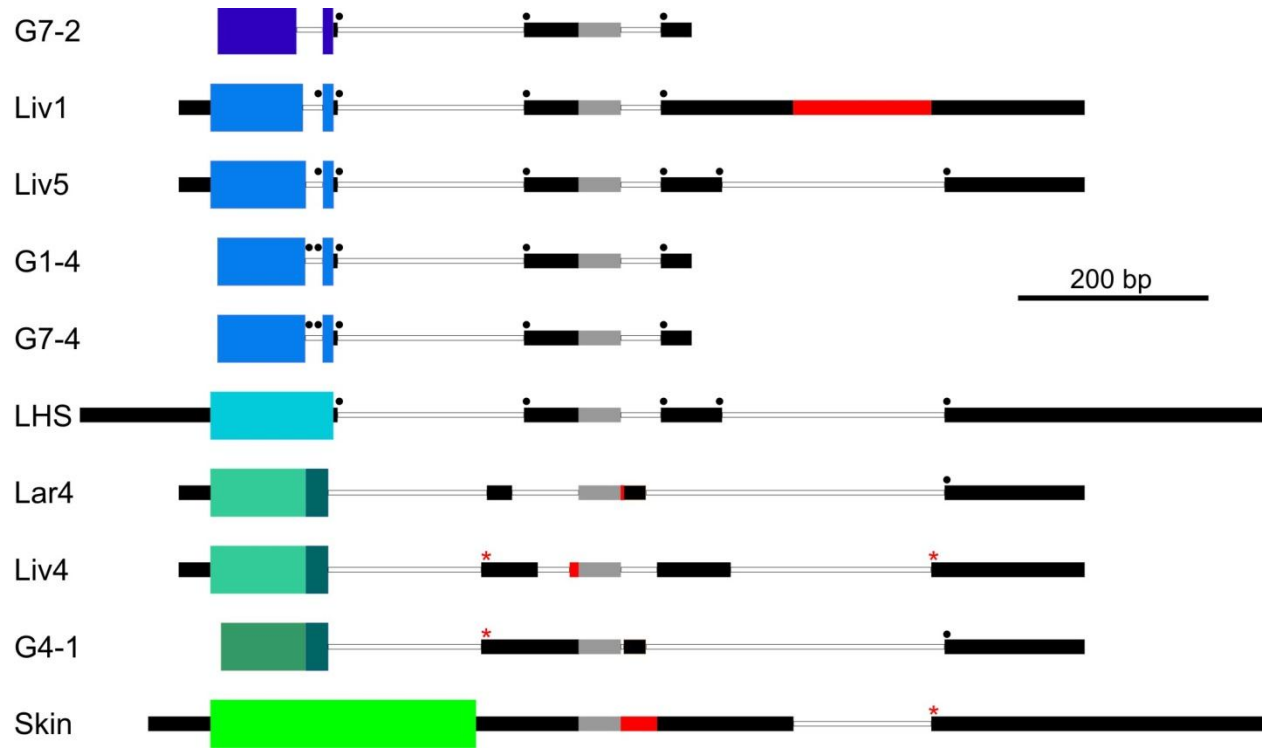

Supplement: Figure S2 — Comparative schematic of sculpin type I AFP genes. A total of eight new sequences are shown after those with three or fewer silent or non-coding mutations were exuded. They were obtained from cDNAs isolated from liver (Liv) or larvae (Lar) or from genomic DNA (G) and are compared to known shorthorn skin (Skin) and longhorn skin (LHS) sequences. Coding sequences are shown by thick bars, with color gradation approximating relative similarity. Gaps are indicated by hollow thin bars and non-coding sequence by filled bars of intermediate thickness. Gray bars indicate a hypervariable region, containing GTn and Gn repeats, from which deletions and fine detail has been omitted. Red bars indicate unique sequence present in but a single clone. Identical symbols (red asterisks or black circles) indicate shared breakpoints. (PDF) [file pone.0081285.s002.pdf]

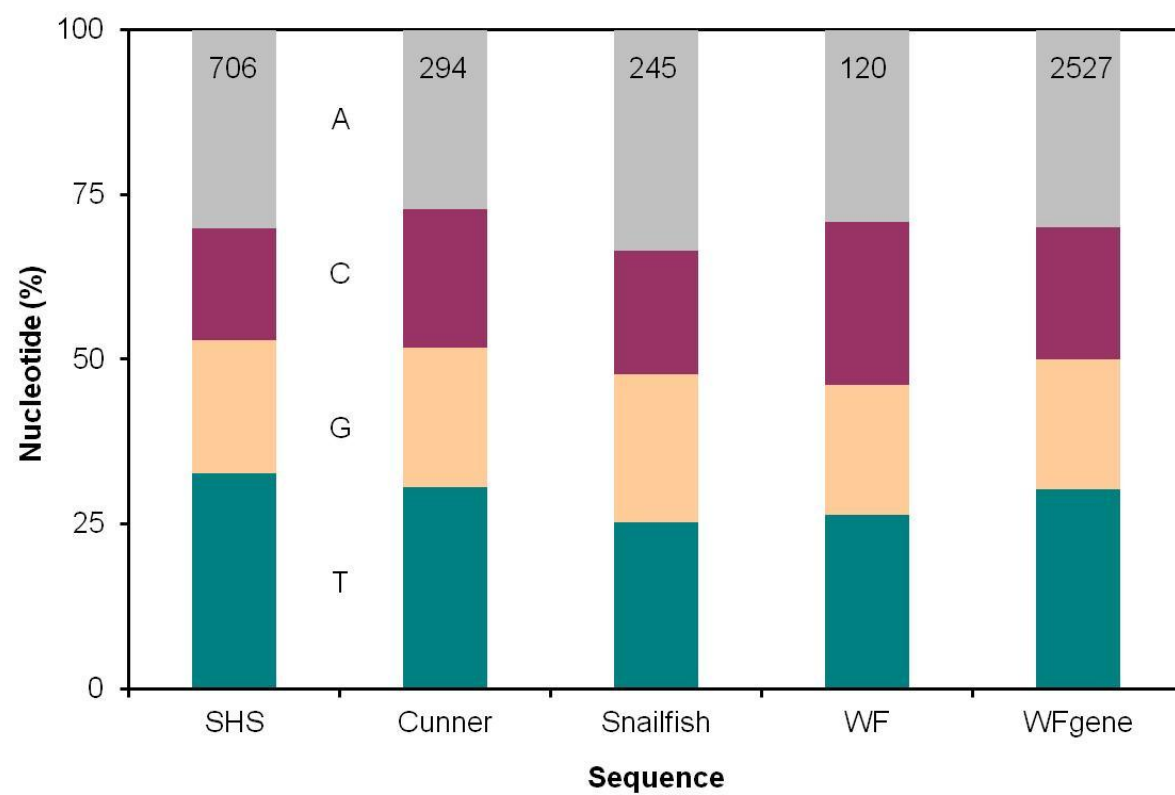

Supplement: Figure S3 — Nucleotide content of flanking non-coding regions of selected type I AFP cDNA sequences. The sequences used are as follows; shorthorn sculpin (skin, AF305502.1), cunner (JF937681.2) and snailfish (AY455863.1). The HPLC6 gene of winter flounder (M62415.1) is also shown in which the UTRs (WF) are scored separately from the intronic, upstream and downstream sequence (WFgene). The total number of nucleotides scored for each is indicated in the gray bar. (PDF) [file pone.0081285.s003.pdf]

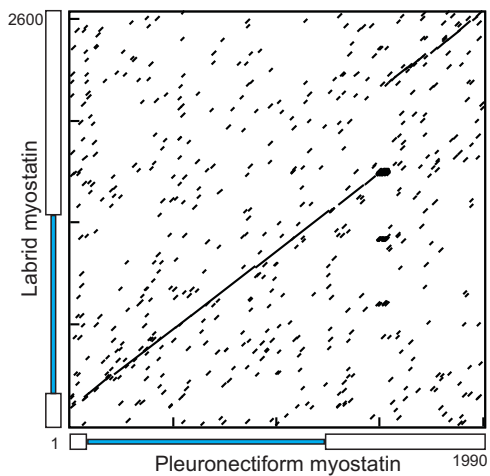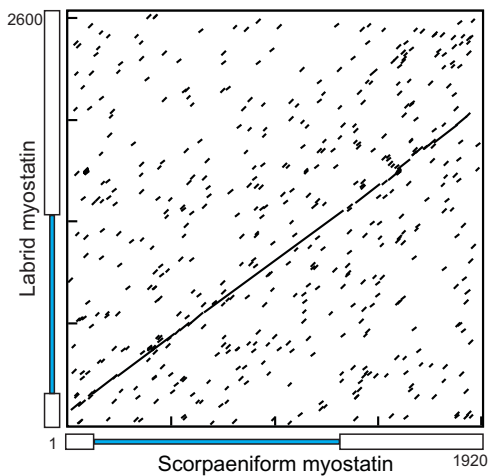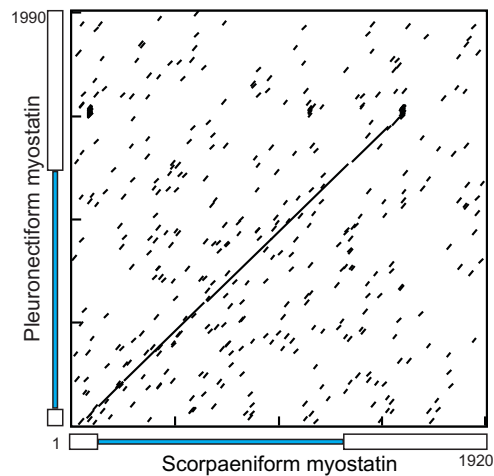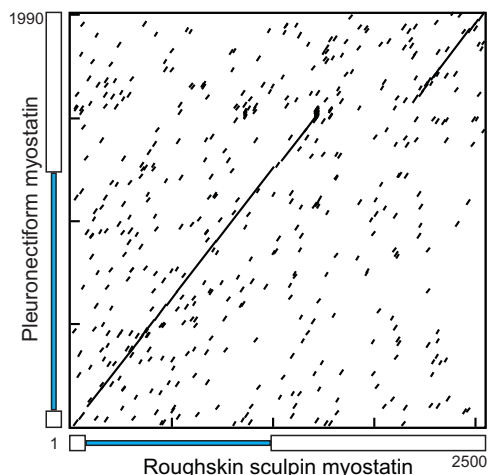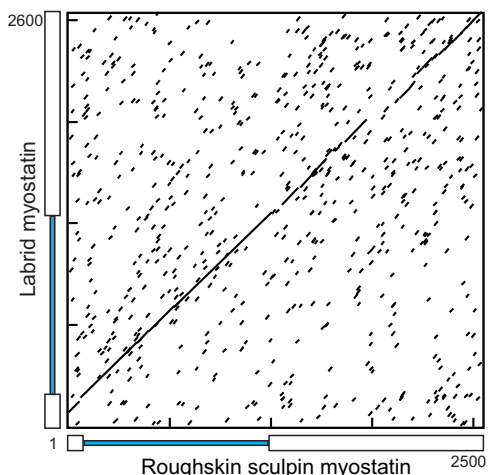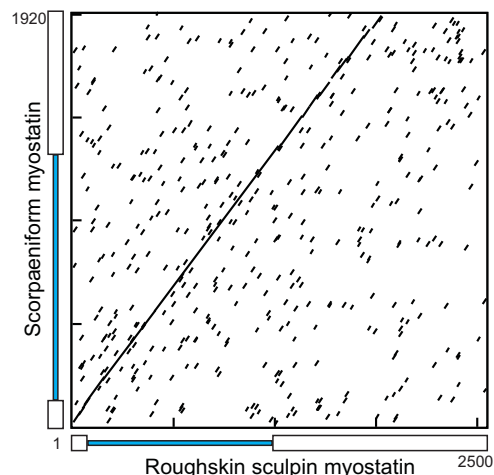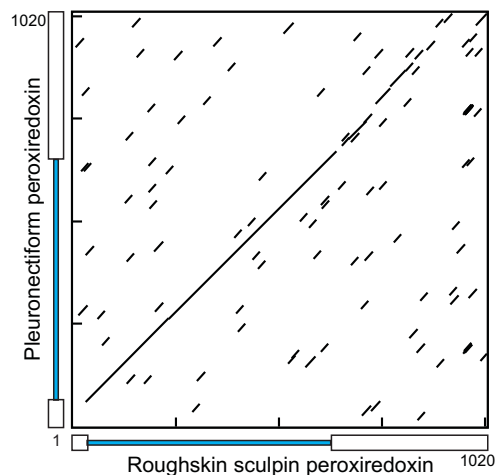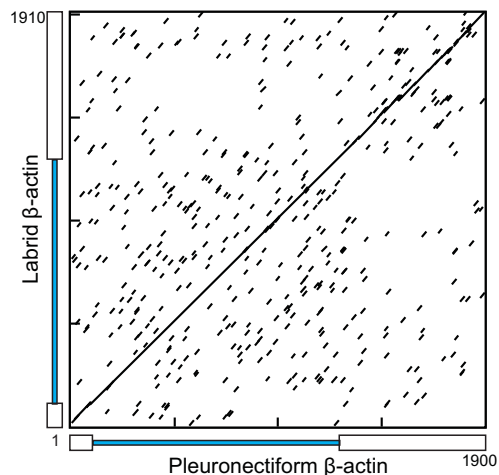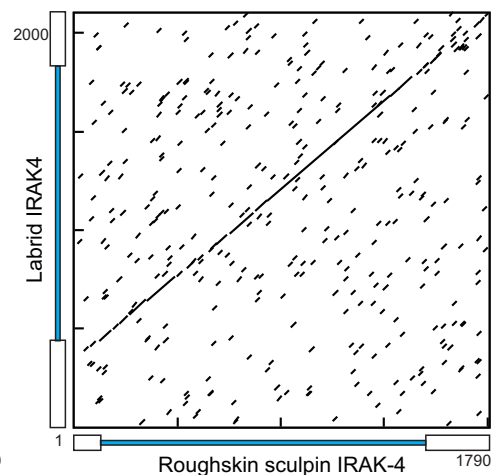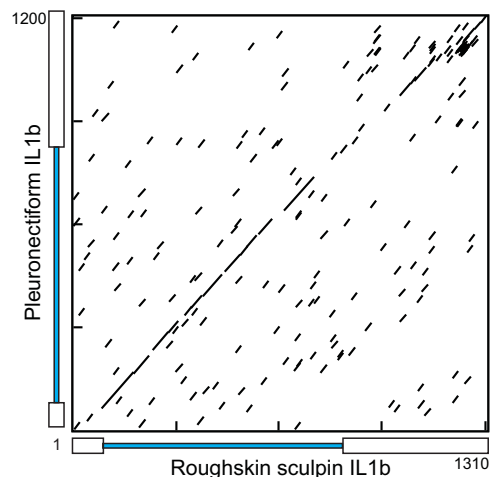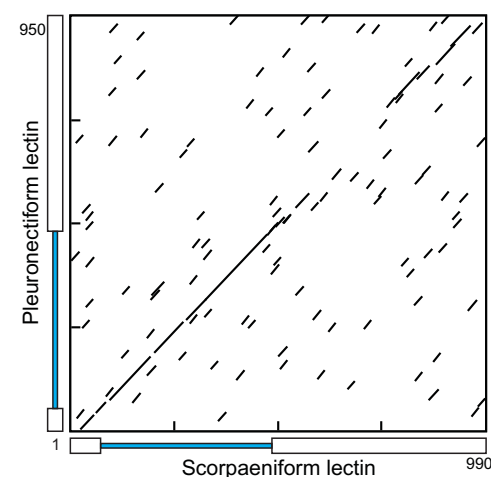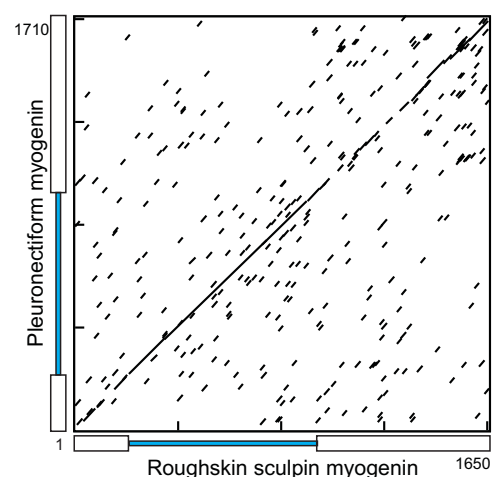

Supplement: Figure S4 — Dot matrix comparisons of non-AFP cDNAs from the four different groups of fishes. A line indicates a match of at least 9 out of 12 bases. Antisense matches were excluded for clarity. The thin blue bars denote the coding region with thicker hatched yellow bars denoting the untranslated regions. Homologs to 10 of 14 protein-coding non-mitochondrial cDNAs from the roughskin sculpin, Trachidermus fasciatus, were found in the non-redundant (nr) database within one or more of the type I AFP producing fish groups (Fig. 1); Labridae (cunner), Pleuronectiformes (flounder) and suborders of Scorpaeniformes external to both sculpins and snailfish. Homologues to three cDNAs were found in all groups and pairwise comparisons are shown for myostatin, with a single comparison to the most divergent group (Pleuronectiformes) shown for peroxiredoxin and β-actin. Four additional comparisons, selected from the seven other groups of homologs, are also shown. The most divergent UTRs (IRAK-4) can be contrasted with the least convergent (β-actin). The GenBank accession numbers for the sequences compared (those used in the figure are bolded, with the gene name as it appears in the figure in italics) are as follows; myostatin GU198192.1 (sculpin), DQ423474.1 (scorpaeniform), XM_003458832.2 (labrid), EU443627.1 (pleuronectiform); β-actin HM449124.1 (sculpin), JN226153.1 (scorpaeniform), XM_005743477.1 (labrid) HQ386788.1 (pleuronectiform); peroxiredoxin-1 JQ911738.1 (sculpin), AB490894.1 (scorpaeniform), XM_003453360.2 (labrid), DQ009987.1 (pleuronectiform); galactoside-binding lectin JX908825.1 (sculpin), BT082644.1 (scorpaeniform), DQ993254.1 (pleuronectiform); myogenin JQ905626.1 (sculpin), XM_005739439.1 (labrid), EF144128.1 (pleuronectiform); interleukin 1-β (IL1B) JQ319051.1 (sculpin), AB491084.1 (scorpaeniform), FJ769829.1 (pleuronectiform); interleukin-1 receptor-associated kinase 4 (IRAK-4) JQ319050.1 (sculpin), XM_003443911.2 (labrid), FJ825148.1 (pleuronectiform); thioredoxin domain [file pone.0081285.s004.pdf]
