# Supplementary material for: Helical Antifreeze Proteins Have Independently Evolved in Fishes on Four Occasions
Source: PLoS One. 2013 Dec 6;8(12):e81285. doi: 10.1371/journal.pone.0081285 (PMC3855684; doi:10.1371/journal.pone.0081285)
Supplement: Text S1 — Accession numbers of coding sequences used to calculate codon usage frequencies. The non-AFPs are listed first with the total number of sequences used indicated. The accession numbers of the AFPs are listed last. (PDF) [file pone.0081285.s005.pdf]

Winter flounder (*Pseudopleuronectes americanus*) 70
AF012462.1
AF012463.1
AF012464.1
AF012465.1
AF043384.1
AF102261.1
AF137182.1
AF156788.1
AF156789.1
AF156790.1
AF252633.1
AF301506.1
AF301511.1
AF301513.1
AF369067.1
AF512561.1
AF512562.1
AY053461.1
AY156726.1
AY174870.1
AY225096.1
AY225097.1
AY225098.1
AY225099.1
AY225101.1
AY225103.1
AY225104.1
AY521655.1
AY521656.1
AY521657.1
AY521658.1
AY521659.1
AY521660.1
AY521661.1
AY521662.1
AY521663.1
AY521664.1
AY521665.1
AY521666.1

AY521667.1
AY521668.1
AY521670.1
AY521671.1
AY521672.1
AY631037.1
AY631038.1
AY631039.1
AY794223.1
AY836578.1
EU684053.1
EU684055.1
FJ379291.1
FJ870490.1
GQ397970.1
GU828013.1
GU828014.1
HQ406771.1
HQ406772.1
HQ406773.1
HQ406774.1
HQ623429.1
HQ623430.1
HQ659503.1
L11615.1
U03674.1
U13963.1
U45877.1
X13594.1
X74492.1
Z97028.1

Longhorn sculpin (*Myoxocephalus octodecemspinosus*) 10
AF026525.1
DQ520199.1
EU391598.1
EU391599.2
EU391600.1
EU444011.1
EU440324.1

EU703783.1
EU909191.1
JN252127.1

Liparids 8
AY362235.1
AY539253.1
AY539452.1
AY547502.1
AY547503.1
AY547504.1
DQ168081.1
EU492247.1

Cunner (*Tautogolabrus adspersus*) 10
AY279855.1
AY279958.1
AY496969.1
GU596480.1
GU596481.1
GU596482.1
HM050022.1
HM050081.1
HM050201.1
HM050264.1

Winter flounder hyperactive AFP
EU188795.1

Longhorn sculpin skin AFP
AF306348.1

Shorthorn sculpin skin AFP
AF305502.1

Atlantic snailfish AFP
AY455862.1

Cunner AFP
JF937681.2
